# Neuroendocrinology and its Quantitative Development: A Bioengineering View

**DOI:** 10.1186/1475-925X-9-68

**Published:** 2010-11-04

**Authors:** Max E Valentinuzzi

**Affiliations:** 1 Instituto de Ingeniería Biomédica (IIBM), Universidad de Buenos Aires (UBA), Paseo Colón 850, 4°p, (1063) Ciudad de Buenos Aires, Argentina

## Abstract

Biomedical engineering is clearly present in modern neuroendocrinology, and indeed has come to embrace it in many respects. First, we briefly review the origins of endocrinology until neuroendocrinology, after a long saga, was established in the 1950's decade with quantified results made possible by the radioimmunoassay technique (RIA), a development contributed by the physical sciences. However, instrumentation was only one face of the quantification process, for mathematical models aiding in the study of negative feedback loops, first rather shyly and now at a growing rate, became means building the edifice of mathematical neuroendocrinology while computer assisted techniques help unravel the associated genetic aspects or the nature itself of endocrine bursts by numerical deconvolution analysis. To end the note, attention is called to the pleiotropic characteristics of neuroendocrinology, which keeps branching off almost endlessly as bioengineering does too.

## 1-Introduction

Scientific disciplines constantly evolve, usually starting at a *qualitative stage *(by being mostly *descriptive*, as the early anatomical or zoological knowledge was), to enter later on into more *quantitative stages *(like counting the number of lobes of an organ). Obviously, some disciplines are more *quantifiable *and *quantified *than others. The cardiovascular and respiratory systems, for example, are easier in this respect because their variables (such as pressure and flow) have precise mathematical definitions. Psychophysiology, instead, does not have yet clear-cut variables and, as a consequence, its quantification process is slower [1]. In such line of thought, the intent here points out to the growing quantitative and manifold characteristics of modern neuroendocrinology, quite similar to those of bioengineering, so much, that the latter interdiscipline now also embraces the former in many respects.

Neuroendocrinology, as a well defined but now separate area of endocrinology, is still relatively young. Its roots can be traced back to the French scientist Claude Bernard (1813-1878) with his studies on pancreas and also even laying the foundations for the study of molecular signaling in endocrinology [2,3]. Bernard already appreciated the **importance of mathematics and stated that their application to natural phenomena is the aim of all science**; however, he also believed that many attempts were faulty because empirical data were insufficient [4]. Charles Edouard Brown-Sequard (1817-1894), Bernard's student and a controversial man in some respects, was one of the first to postulate the existence of substances, now known as hormones, secreted into the bloodstream to affect distant organs [5]. In particular, he demonstrated (in 1856) that removal of the adrenal glands resulted in death [6]. However, endocrinology's birth certificate was issued with the demonstration by William Bayliss and Ernest Starling, in 1902, that acid liberates a chemical messenger (secretin, a 27 amino acid peptide) from the cells of the duodenal and jejunal mucosa and that this, via the blood stream, excites the pancreas to secrete juices [7].

In the 1930's, the hypothalamic control of the pituitary gland was pretty much accepted; however, it took several years until Ernst Scharrer, in 1945, and his wife Berta [8] showed that the preoptic region of the brain has endocrine properties associated with pituitary function. Meanwhile, two other important and related concepts were being developed: Walter Bradford Cannon (1871 - 1945) coining as early as 1925 the terms **fight or flight**, obviously anticipatory of the stress idea, to describe an animal's response to threats (as *body changes in pain, hunger, fear and rage*), and by the introduction of another word, **homeostasis**, stemmed clearly in Claude Bernard's idea of ***milieu interieur ***(internal environment) widely popularized in his book *The Wisdom of the Body *(1932). Along with this and as early as 1926, when still in his second year of medical school, Hans Selye (1907-1982) began developing his theory of the influence of stress on a person's ability to cope with and adapt to injury and disease. He found that patients with a variety of ailments manifested many similar symptoms, which he ultimately attributed to their bodies' efforts to respond to the stresses of being ill [9-12].

Ideas, experimental data, and knowledge were quickly growing and ripening. Towards the end of the 1950's, Roger Guillemin (Selye's former student) and Andrew Schally, in their respective laboratories, were able to extract from the hypothalamus of sheep and pigs compounds which, when administered to adenohypophyseal tissue, brought about release of its hormones. One extract triggered the pituitary release of ACTH (Adreno-Corticotropic Hormone), another was linked to TSH (Thyroid Stimulating Hormone), still a third was stimulus for the secretion of LH (Luteinizing Hormone) and a fourth was it for the secretion of FSH (Follicle Stimulating Hormone), the latter two collectively named gonadotropic hormones. They termed these hypothalamic substances *releasing factors *or *releasing hormones*, RF or RH, so that, for example, the one inducing the release of TSH, was called TSH-RF or TRF. Quite significant were the many contributions in the area; referring specifically to them exceeds the limits of this note (see, for example, the following websites, http://www.nobelprize.org/nobel_prizes/medicine/laureates/1977/schally-autobio.html, or http://www.faqs.org/health/bios/51/Roger-Guillemin.html).

^A caveat that may lead to misnomering: Gonado**tropic **or luteo**tropic **or any other terms with the same endings derive their suffix **tropic **from Greek, *trepein*, "to turn into", as a phototropic plant, because it turns to light. Do not spell "gonadotrophic" because *trephein*, also from Greek, means to feed or to nourish, giving the idea of growth, as "hypertrophy" or its opposite "atrophy" (lack of growth).^

It was not until 1969 that the nature of these hypothalamic factors would be established. Guillemin was working with 5 million hypothalamic fragments from sheep, and Schally with the same amount of material but from pigs. They concentrated their efforts to the searching of one releasing factor. After a long struggle, they obtained 1 mg of a pure substance, TRF, with a single action: release of TSH from the hypophysis. Soon theresafter, the structure of TRF was established (it is an extremely small peptide composed of three amino acids), no doubt, a quantitative step forward. Within the same year, TRF was synthesized by Guillemin's group and two years later LH-RH was isolated, sequenced and synthesized, another highly significant and important quantification step, first by Schally and shortly afterwards by Guillemin. Experience in animal research was rapidly transferred to humans and brought into clinical work. Several new peptides were isolated from the hypothalamus, the foremost one probably being the first inhibitor of pituitary function, somatostatin, which decreases the production of pituitary growth hormone. In 1977, Andrew Schally and Roger Guillemin were awarded the Nobel Prize, along with Rosalyn Sussman Yalow, for their discoveries concerning **the peptide hormone production of the brain **http://nobelprize.org/nobel_prizes/medicine/laureates/1977/.

But what was Rosalyn's contribution? What other factors converged to the outstanding results and success? She is a physicist and mathematician, hence with a background not related to the biomedical sciences. Solomon Berson, her close collaborator, was a physician. Getting acquainted with their lives provides surprising and touching human insights. Radioimmunoassay (RIA), developed by them, allowed precise and minute concentration measurements of a substance, by quantitating the binding, or the inhibition of binding, of a radiolabeled substance to an antibody [13-15]. It was a revolutionary highly quantified diagnostic process that was largely ignored when Yalow and Berson published it in 1960. Their primary use was intended for the study of diabetes but today its applications are multiple. Yalow and Berson (the latter died prematurely in 1972) could have patented RIA and would have gotten enormous royalties. Instead, to their credit, they made efforts to get RIA into common use; they wanted its potential value directed to humankind ahead of its potential value to themselves. Quite a human lesson to underline with not too many examples to mention. Thus, neuroendocrinology's final birth certificate, based on strong and well-behaved numerical grounds and, why not, we may add **with contributions from bioengineering**, can be established between the late 1950's and early 1960's.

The qualitative stage has been accomplished (that is, descriptions, cause-effect relationships, pathways of action, targets). Say, well established are the higher central nervous system links to the adenohypophysis, in turn aimed at different targets involving also negative regulatory feedback loops. The identified measurable variables are action potentials at the neural side, carrying information perhaps as frequency-like modulation, and exquisitely controlled concentration levels at the vascular side. Obviously, electronic instrumentation advancements (such as microelectrode techniques brought forward from the electrophysiology saga) played a significant role in the former. For example, goldfish hypothalamic neuroendocrine cells have been investigated with intracelular recordings showing resting potentials of 50 mV and action potentials up to 117 mV followed by a long lasting and prominent diphasic hyperpolarizing after-potential. Neuron input resistance was found in the order of 3.3 × 10^7^Ω with a total time constant of 42 ms [16]. No doubt, good quantitative information.

In the meantime, some people analyzed the feedback loops from the control theory viewpoint, trying to quantify them, discussing the linear versus the non-linear approaches and bringing about mathematical models [17,18]. But there is more recent news, such as the hypothalamic-pituitary-thyroid axis model proposed by Liu *et al*, in 1994 [19], from China, who took into account both the binding of hormones with proteins in the plasma and tissues and the interactions of the hormones in the axis. Results calculated from the model were in good agreement with experimental data. Another theoretical approach deal with prolactin rhythm in rats [20] while there is a relatively recent more general treatment by Leng and MacGregor [21], where the authors introduce some styles of modelling as they have been applied to neuroendocrine systems and discuss some of their strengths and limitations. This editorial is not a review and, regarding this subject area, we only add that a workshop on Mathematical Neuroendocrinology was organized by the Mathematical Biosciences Institute of Ohio State University (August 9-13, 2010; http://mbi.osu.edu/2010/mndescription.html).

Sequencing of the human genome certainly greatly influenced the quantification process, where computer techniques act as essential aid. Obviously, all this encompassed a rather significant advance as concrete well-defined numbers started to enter the picture. The development of mathematical models leads to *prediction*, which is the highest level of quantification and, after all, what is a physician trying to do when he/she examines a patient? To determine what the disease is and, above all, to **predict its most probable course**. A veterinarian, an ecologist and other biologically related activities take a similar stand. In short: Scientific disciplines show a slow, sometimes faster, but steady process of quantification. Biology, physiology and medicine are no exceptions. A still distant and well-yearned objective is to anticipate disease, as much in advance and as much quantifiably as possible, based on the current known condition of a given individual. For the time being, even with the tools nowadays at hand of the medical profession, that prediction is still far from being exact and precise.

## 2-Characterization of a system

Control engineers are familiar with the convolution integral: In a linear system, knowing the input signal and the function describing such system, both as time dependent events, can lead to the system's response by convolving one with the other. A mathematical operation called *convolution *is required. Its inverse operation is *deconvolution*, i.e., knowing say the input and the output functions can produce the function that characterizes the system. Neither of these operations is an easy task, especially the second one. More than that, in some fields (as physiology), the explicit mathematical functions are never known and we only have in our hands discrete experimental data points that eventually or often do not abound. To complicate further the overall scenery, linearity shows up as a big and many times highly debatable IF since most of biological processes rarely, if ever, behave in a linear way.

Notwithstanding the abovementioned difficulties, deconvolution has been used with considerable success by Segre to characterize the dynamics of intestinal absorption as early as 1967 [22]. Moreover, years later, several authors carried out a similar approach to quantitatively uncover the renal retention function [23-28]. And the turn arrived in 1987 for neuroendocrinology by using a simple, ingenious and clever idea. Traditional control engineering and the two previously referred to physiological applications of deconvolution analysis require the injection of a known input signal, which by and large is the impulse delta Dirac function (any other could be employed just as well, but the delta function has some advantages). Such function, usually represented by δ(t), was introduced by Paul Adrien Maurice Dirac (1902 - 1984), a British theoretical physicist; it takes the value infinite, **∞**, at time t = 0, and the value 0 for any other instant *t*. Obviously, in practice, such behavior is not truly realizable and can only be approached by large and very short pulses. A salient highly significant feature is that when δ(t) is applied to a linear system, the response read at its output describes the characteristic function of the system, usually termed h(t) and called the *impulse response *of the system. That is what was done when intestinal absorption and renal function were studied, which thereafter, by proper block diagram and deconvolution analysis led to the determination of the system's dynamic constants [22,29].

Several neuroendocrine glands secrete their hormones as short bursts, as well described in many papers, and such bursts can be looked at as physiological imperfect delta functions. The brilliant concept brought forward by Veldhuis, Carlson and Johnson, in 1987 [30], simply said, no need of an external generator or injection for the system has its own. Deconvolution did the rest and a series of superb contributions greatly improved the quantification neuroendocrine process [31].

However, quantitative neuroendocrinology is by far much more than deconvolution. Perhaps, Johnson and Velhuis [31] well characterized the whole intent in the preface of their outstanding treatise, which no doubt we dare qualify as within a clear bioengineering framework. Interspersed quoting from it is appropriate: "*As experimental strategies have become more sophisticated, high-speed computing has been required for the formulation and solution of more elaborate statements of neuroenocrine pulsatility, such that matrices are needed to handle 100 to 300 equations, each containing 10 to 30 variables. Obviously, neuroendocrinology in its quantitative endeavor appears as a multidisciplinary entity with outstanding contributions from probability theory, systems engineering, stochastic differential equations and the experimental natural sciences such as cell and molecular biology and other approaches to subcellular analyses*". Five out of sixteen chapters (that is, 31% of the whole book) deal with deconvolution analysis based on the burst intrinsic glandular secretion, as the delta function simil or S(t), the blood hormonal concentration C(t) as the output signal, and the system elimination function E(t), as the characteristic time transference function called h(t) in systems engineering, meaning the importance that these editors deem the subject.

## 3-New quantitative and pleitropic findings

The last decade (200-2010) has witnessed surprising and outstanding news that make us wonder where the limits of neuroendocrinology really stand, something that make us think of its pleiotropic functions or pleiotropic characteristics or, in short, the pleiotropism of neuroendocrinology (the term *pleiotropy *comes from the Greek πλεíων or *pleion*, meaning "more", and τρέπειν or *trepein*, meaning "to turn, to convert"; see also the note in the first section). Thus, pleiotropic functions refer to producing more than one effect, as a pleiotropic gene, which has multiple phenotypic expressions because it has signaling function on various targets, because it branches off. Let us briefly review just a few recent examples reported in the literature.

Douglas E. Brenneman, Joanna M. Hill, and Illana Gozes, in 2000 [32], referred to the vasoactive intestinal peptide (VIP), which is a multi-functional neuropeptide with roles that extend far beyond actions in the small intestine (where it was originally isolated). Since its discovery in 1970, over 7000 papers have been written on VIP (as some kind of Very Important Person, turning jokingly into a common use of the acronym), with 16% of these referring to the brain. VIP is a 28-amino acid peptide that is widely distributed in the central and peripheral nervous systems. Important actions for VIP have been reviewed for the cardiovascular, reproductive, pulmonary, immune and gastrointestinal systems. General physiological effects include vasodilation, bronchodilation, immunosuppression, hormonal secretion and increases in gastric motility. However, there are also contributions related to gene expression, receptor characterization, drug design, functional neuroanatomy, neuroendocrine regulation, growth regulation and clinical pathology, therapeutics and even quite attractive concepts as neurotrophism without neurotropism, where the brain derived neurotrophic factor (BCNF) is involved (see Lu *et al*) [33], the latter authors from San Diego, La Jolla, CA.

Galanin-like peptide (GALP) is a newly discovered hypothalamic neuropeptide, which is regulated by leptin and implicated in the regulation of GnRH (Gonadotropin-releasing hormone, also known as Luteinizing-Hormone-Releasing Hormone or LHRH), the latter responsible for the release of FSH and LH from the anterior pituitary. After searching the human genome database it was determined that the human GALP gene comprises six exons. Mature GALP is predicted to be 60 amino acids in the macaque as in other species. Besides, the distribution of GALP mRNA in the hypothalamus and pituitary of the macaque showed that, as in rodent species, the expression of GALP mRNA is confined to the arcuate nucleus, median eminence, and neurohypophysis [34].

Matthias Tschöp and Tamas L. Horvath [35] commented the increasingly sophisticated methods that have been brought to bear on the problem of the brain involvement in the physiology of energy homeostasis and the pathogenesis of obesity. A vast number of experimental observations have been produced. The combination of genetic and sophisticated physiology techniques has allowed for great progress. These methods helped the identification of metabolic hormones and their relationship to key peptidergic systems in the hypothalamus. Furthermore, researchers are far from yet understanding the overall picture of central body weight regulation that involves multiple brain areas outside the hypothalamus.

Brighton, Szekeres and Willars [36] studied Neuromedin U (NmU). It is a structurally highly conserved neuropeptide. It is ubiquitously distributed, with highest levels found in the gastrointestinal tract and pituitary. Originally isolated from porcine spinal cord, it has since been isolated and sequenced from several species. Amino acid alignment of NmU from different species reveals a high level of conservation, and particular features within its structure are important for bioactivity. The conservation of NmU across a wide range of species indicates a strong evolutionary pressure to conserve this peptide and points to its physiological significance. NmU was first isolated based on its ability to contract rat uterine smooth-muscle (hence the suffix "U") and has since been implicated in the regulation of smooth-muscle contraction, blood pressure and local blood flow, ion transport in the gut, stress responses, cancer, gastric acid secretion, pronociception, and feeding behavior.

Ying Yang, Li-bin Zhou *et al *[37] investigated the expression of feeding-related peptide receptors mRNA in GT1-7 cell line and roles of leptin and orexins in the control of GnRH secretion. It was concluded that feeding and reproductive functions are closely linked. Many orexigenic and anorexigenic signals may control feeding behavior as well as alter GnRH secretion through their receptors on GnRH neurons.

Glucagon-like peptide-1 (GLP-1) promotes glucose homeostasis through regulation of islet hormone secretion, as well as hepatic and gastric function. Because GLP-1 is also synthesized in the brain, where it regulates food intake, it is hypothesized that the central GLP-1 system regulates glucose tolerance as well, are concepts put forward by Sandoval *et al *and Lauffer *et al *[38,39].

## 4-Conclusions

All these contributions brought quantification to neuroendocrinology, and this is precisely one of the general objectives of bioengineering (or biomedical engineering): to bring quantification into the biological sciences or at least to show pathways leading to it. Experimental methods and techniques encompass one of the earliest and most obvious ways, but uncovering a possible mathematical description, especially when feedback and feedforward loops are quantitatively included (as for example the feedback constant, often called β in the engineering jargon) represents a quantitative step forward. Disclosing the molecular structure of a hormone, with its molecular weight and other related parameters, its affinity with other biochemical structures (as radicals may be), identification of receptor sites, gene expression, and the like are other quantifying forms. Most important and not fully known yet appear the transduction mechanisms linking neural action potentials, -modulated in frequency or in some form of pulse position-like modulation to carry on stimulating or inhibiting information- to actual secretory gland activity measured in micro or nanograms per unit time, which in the end will mean hormonal concentration in blood expressed in mass units per milliliter. Do we have an efficiency factor for those transducers, that is, a parameter saying that so many action potentials are required to secrete so many nanograms of hormone in one second? Predictive mathematical models are still quite ahead in our dreams although some pathways are being opened while a technological demand unit instructing the gland to increase or decrease its amount of secretion does not appear as too far fetched. No doubt, neuroendocrinology entangles with and forms part of bioengineering while the Seven Lamps of the latter keep spreading their lights [40].

## Note

To measure is to know, if you can not measure it, you can not improve it; after all, there is no real satisfaction in formulas alone unless you can feel their numerical magnitude. Sir William Thomson, Lord Kelvin (1824-1907), British Physicist and Mathematician
